# Professional's progress: Learning from life and mistakes

**DOI:** 10.4103/0019-5545.31557

**Published:** 2006

**Authors:** C. Shamasundar

**Affiliations:** * Retired Professor of Psychiatry, NIMHANS, Bangalore, 250, 43rd Cross, 9th Main, 5th Block, Jayanagar, Bangalore 560041, Karnataka; e-mail: drshamasundar@gmail.com

## BACKGROUND

The scope of the topic indicated in the title is as vast as one's life. In addition, a large proportion of learning will have happened outside the participation of one's awareness. Among those events that prompted conscious learning, many would have been lost to memory, and some of them would be too private to communicate.

Learning and growth in one's life, either personal or professional, is an inevitable process. However, the issue or question about their mutual relationship has enjoyed a relative neglect. Many of the important lessons that I learnt have already been communicated in my two previous articles.^1^,^2^ I am narrating here a few more items of learning that I happen to remember.

### Few initial clarifications

According to ancient Indian tradition, there are many pathways to learning. But, experiential learning claims superiority. Another important mode of learning is by trial-and-error. There is a proverb in Kannada, which hints at both, *Oni kusu beleyitu, kone kusu koleyitu.* Translated into English, it means: ‘a baby brought up on the street survived, and the one brought up in a room did not’. In this context, learning and closely related issues need some brief descriptions. Generally, learning is taken for granted. But, it is not so simple, nor is it complicated. Any goal-directed behaviour is a result of executing a decision. The making of a decision (also called ‘exercising an option’ or ‘making a choice’) is based on a set of constructs, concepts or assumptions.

Learning, either useful or harmful, takes place when a result of behaviour influences one's ‘world-view’ (or, ‘assumptive world’). The process by which this influence takes place is ‘experience’. This experience becomes the basis of ‘experiential learning’. A consequence of this experience is: some of the initial concepts or assumptions, or their opposites or modifications become part of one's values and belief-systems, one's world-view. Decision-making, mentioned earlier, always involves some degree of judgements and compromises. Thus, decisions are subject to what later may get considered as errors or mistakes. This potential for errors or mistakes is further compounded by a degree of inherent uncertainty of outcome. These mistakes become the basis of ‘trial-and-error learning’ at best, and of regretful, self-blaming distress at worst, depending upon how an individual responds to failures.

### Examples of two types of learning

I will now narrate two stories to demonstrate experiential and trial-and-error learning respectively. The first is a folk story from the Indian Epic *Mahabharata.*^3^ Prince Yudhisthira had started his studies in a reputed school. One day, the teacher dictated to the class ten principles to be learnt by students. Next day, he asked the students to report one-by-one what each had learnt. Each student dutifully recited the ten principles that had been committed to memory. When his turn came, Yudhisthira said he had learnt the first principle, and was still learning the second one. At this, the teacher who had expected a better performance from the prince became angry and started beating him with his cane. But, the Prince stood calm and composed. Seeing his composure, the teacher suddenly became aware that some mistake may have occurred and thought, ‘….this future King, who can get me executed by a mere gesture, is so composed in spite of my beating….He says he has learnt the first principle and still learning the second one… What are they?… The first one is “always tell the truth,” and the second one is “control your anger”….’ Immediately, the teacher realized what the Prince meant by learning a principle. For him, learning a principle meant becoming a living example of what it says! Overwhelmed by joy, he embraced him, sought his forgiveness, and said, ‘You said you are still learning the second principle. But, you have already mastered it’.

Yudhisthira replied, ‘No sir, I have not yet mastered it. When you were beating, anger was raising in me, and I was struggling very hard to control it.’

The second story happened in real life in the eighties when I visited Ahmedabad with my colleagues to participate in the annual conference of the Indian Psychiatric Society. We had an invitation from an acquaintance to visit his wholesale *saree* shop one evening and have dinner with him.

In the *saree* shop he introduced his two sons, ‘….this is my first son. I have trained him in business, and he is looking after half of it. This is my second son. I am training him. When he completes his training, I will hand over the other half and retire….’

Having been used to training our residents with structured programme and assessing their learning by structured method, I became curious to know: How does this business man train his sons, and how does he know when the training is complete? During dinner, I asked him.

He replied, ‘I send them to buy *sarees* from different manufacturers in Kanchi, Coimbatore, Calcutta, Varanasi, etc. I similarly send them for collecting the arrears of credits we have given the buyers. And, I will put them at the sales counter….’

I said, ‘This is how you train them, OK. But, how do you know when the training is completed?’

He replied, ‘I know they are trained when they incur a loss of one or two hundred thousand rupees’.

I was aghast and asked him, ‘What has this loss got to do with training?’

He explained, ‘When they lose a lakh or two, they will have learnt whom to trust and whom not to trust with credits, what designs sell and what does not, and the trend of the fashion…..’

This is an example of giving responsibility to foster trial-and-error learning. This narrative explains one of the meanings of the Kannada proverb quoted earlier. One does not learn by avoiding hardships and failures, but by bravely facing and managing them, being ready to ‘pay the necessary price’.

### Learning from mistakes

Trial-and-error learning involves learning from consequences of one's behaviour. Some of the consequences will be mistakes. It is also logically possible to learn from others' mistakes, provided one knows the constituent inputs that led to a given mistake.

I will now briefly narrate two mistakes from my clinical conduct. In the early years of my career, I was once seeing a depressed young woman for psychotherapy. After three sessions or so, I had felt satisfied with my therapeutic effort. The severity of patient's symptoms had reduced, and I had a fairly neat and detailed psychodynamic formulation to boast about. In the fourth or fifth session, she began to intersperse her narratives with ‘…do you understand?…’ I was irritated by its implication and asked her what makes her doubt that I have not understood. She did not turn up for subsequent appointments. I reviewed my therapy notes (after each session, I used to write down whatever I remembered about the session). I was appalled to notice that in the two previous sessions, I had repeatedly missed her veiled clues about an extramarital affair. There were three lessons for me: (i) At no point in therapy can we ever claim to have completely understood the patient; (ii) We can only understand to the extent that the patient allows us to understand, and to the extent that we alertly catch the clues and hints; (iii) The patient knows whether we have understood or not.

An year or two later, I was given a patient for psycho-therapy; he was a premorbidly well-adjusted middle-aged man having mild depression on account of a persistent stress. I expected him to require just two or three sessions. After four or five sessions, I suddenly became aware that ‘I was stuck with the patient’. When I reviewed my notes, I realized that I was adapting a technique that I did not believe in. I was attempting to practice a technique of repeating the patient's last sentence or phrase. But actually, I had no belief in this technique, which I considered artificial. I gave up that practice, and the patient was discharged from therapy two sessions later. I had two lessons: (i) Patients do not benefit from what we do not believe in; (ii) Patients know when we are pretending. Even though the patient may not be consciously aware of the fact, our pretence will adversely influence patient's responses.

### Professional and personal life—same or different?

Obviously, our learning in personal and professional lives runs parallel. Are they interrelated and complimentary? They ought to be. Otherwise, the dissonance will lead either to a schizoid state (similar to the fictitious ‘Dr Jekyl and Mr Hyde’) or to pretence. Both are counter-productive and very stressful; pretentious life is also very stressful. Such a dissonance, when it exists is possibly a source of severe professional-stress.

Ideally, both professional and personal lives are virtually same. In case they are different by virtue of some degree of cultivated ‘objectivity’, they ought to be complementary. It would be useful to reflect whether we really lose our ‘objectivity’ in our personal lives. My observation is that we do not. Objectivity is always there, either as a consciously pursued orientation in a clinical setting, or as a subconscious ‘vigilante’ in personal interactions. If there is no objectivity, what will be there to learn?

I believe that there are three principles concerning the relationship between personal and professional lives.

1. The first principle is, ‘you cannot give what you do not have’. We have to practice in our private lives what we preach in our professional role. Thus, the two lives telescope into each other. Even though the contexts and circumstances are different, our two lives will be regulated by the same set of values, beliefs, attitudes, behaviour patterns, etc. Otherwise, as I said earlier, our professional behaviour becomes just pretence and counter-productive.
2. The second principle is the fact that problems of life cover the entire gamut of human activity. Hence, in order to be able to effectively empathize with the patient's predicaments, it is necessary for the therapist to be a kind of ‘jack-of-all-trade’, with some minimal degree of wide-based knowledge.
3. The third principle is the natural phases of development. For example, the development of the human psyche, starting with sensory exploration, to differentiation, to de-differentiation or integration. This phase of integration can be considered some thing analogous to the concept of ‘wisdom’. I will attempt to explain one of the examples of this integration. We have so much knowledge available to us, ranging from genetics, neurotransmitters, phenomenology, psychology, nosology, psychotherapy, to psychopharmacology, etc., so many shelves of knowledge. But how many of us have a unifying scheme of harmoniously integrating all our knowledge? I believe that this phase of integration is most difficult. But this is something that every professional must sincerely aspire for.
